# Calcified amorphous tumor of the heart with mitral annular calcification: a case report

**DOI:** 10.1186/s13256-017-1337-9

**Published:** 2017-07-18

**Authors:** Ryo Nakamaru, Hiroki Oe, Katsuomi Iwakura, Takafumi Masai, Kenshi Fujii

**Affiliations:** Department of Cardiology, Sakurabashi Watanabe Hospital, 2-4-32, Umeda, Kita-ku, Osaka, 5300001 Japan

**Keywords:** Calcified amorphous tumor, Mitral annular calcification, End-stage renal disease

## Abstract

**Background:**

Calcified amorphous tumor of the heart is a rare, non-neoplastic cardiac mass characterized by nodular calcium in the background of amorphous degenerating fibrinous material. Clinical diagnosis of calcified amorphous tumor can be difficult, and current single imaging techniques do not specifically differentiate calcified amorphous tumor from other cardiac tumors such as calcified atrial myxoma, calcified thrombi, or vegetation. Complete surgical resection is the treatment of choice for both symptom improvement and prevention of embolization, as well as for pathological diagnosis.

**Case presentation:**

A 70-year-old Asian man with end-stage renal disease complained of chest discomfort during exercise. He had no history of thromboembolism or endocarditis. A transthoracic echocardiogram revealed mitral annular calcification as well as a highly mobile mass (8 × 6 mm) attached to the ventricular side of the posterior mitral valve leaflet. As the mass was highly mobile, suggesting a high risk of embolization, he underwent surgical resection. A histopathological examination revealed multiple nodular amorphous calcifications, along with fibrous connective tissue. There were no identifiable myxoma or malignancy cells. Consequently, the diagnosis of calcified amorphous tumor was confirmed.

**Conclusions:**

In the present case, a calcified amorphous tumor arose from mitral annular calcification. A characteristic of mitral annular calcification-related calcified amorphous tumor is its highly mobile nature, with a high risk of stroke or other systemic embolism. Therefore, surgical therapy should be considered for treatment of calcified amorphous tumors.

## Background

Calcified amorphous tumor (CAT) of the heart is a rare, non-neoplastic cardiac mass characterized by nodular calcium in a background of amorphous degenerating fibrinous material [1]. Although patients are often asymptomatic at presentation, they can present symptoms such as dyspnea, syncope, or central retinal arterial occlusion. These symptoms are caused by flow obstruction or embolization by calcific fragments, and may be fatal [2]. Clinical diagnosis of CAT can be difficult, and current single imaging techniques do not specifically differentiate CAT from other cardiac tumors such as calcified atrial myxoma, calcified thrombi, or vegetation. Complete surgical resection is the treatment of choice for reducing the symptoms and preventing embolization, as well as for pathological diagnosis. The origin and epidemiology of CAT is not fully understood. Approximately 20% of reported cases of CAT were observed in patients with end-stage renal disease (ESRD) [3], which may be related to abnormal phosphocalcic metabolism in those cases [4]. Here we report a case of CAT which may be related to mitral annular calcification (MAC), which was highly mobile, suggesting a high risk of embolization. “MAC-related CAT” should be distinct from other immobile CATs. In addition, a review of the literature of previously reported cases about MAC-related CAT is also included.

## Case presentation

A 70-year-old Asian man with ESRD due to nephrosclerosis complained of chest discomfort during exercise and was referred to our hospital. He had been receiving hemodialysis for 20 years, and had a past medical history of hypertension, hyperuricemia, and reflux esophagitis. He had no history of thromboembolism or endocarditis. He smokes one pack of cigarettes per day, and drinks socially.

He had no significant social and environmental history. His medication list included amlodipine, allopurinol, ferric citrate hydrate, and lansoprazole. He had no family history of cardiovascular diseases.

He was afebrile, with a blood pressure of 114/50 mmHg and a regular pulse rate of 61 beats per minute. His oxygen saturation was 96% on ambient air. There was no evidence of lung rales, cardiac murmur, and abdominal tenderness. His neurological examinations were unremarkable. Blood investigations showed: hemoglobin 14.0 mg/dl, white blood cells 4700/mm^3^, and platelets 134,000/mm^3^. His liver and renal function tests revealed total bilirubin 0.4 mg/dl, aspartate aminotransferase 24 IU/l, alanine aminotransferase 34 IU/l, blood urea nitrogen 54.2 mg/dl, and serum creatinine 11.1 mg/dL. His C-reactive protein was 0.16 mg/dl. His serum electrolyte tests were within normal limits, including calcium and inorganic phosphorus. In addition, his brain natriuretic peptide was raised at 605.1 pg/dl. His blood cultures were negative.

**Fig. 1:**
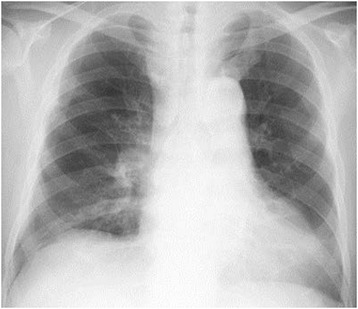
Chest X-ray showing mild cardiomegaly without pulmonary congestion

**Fig. 2:**
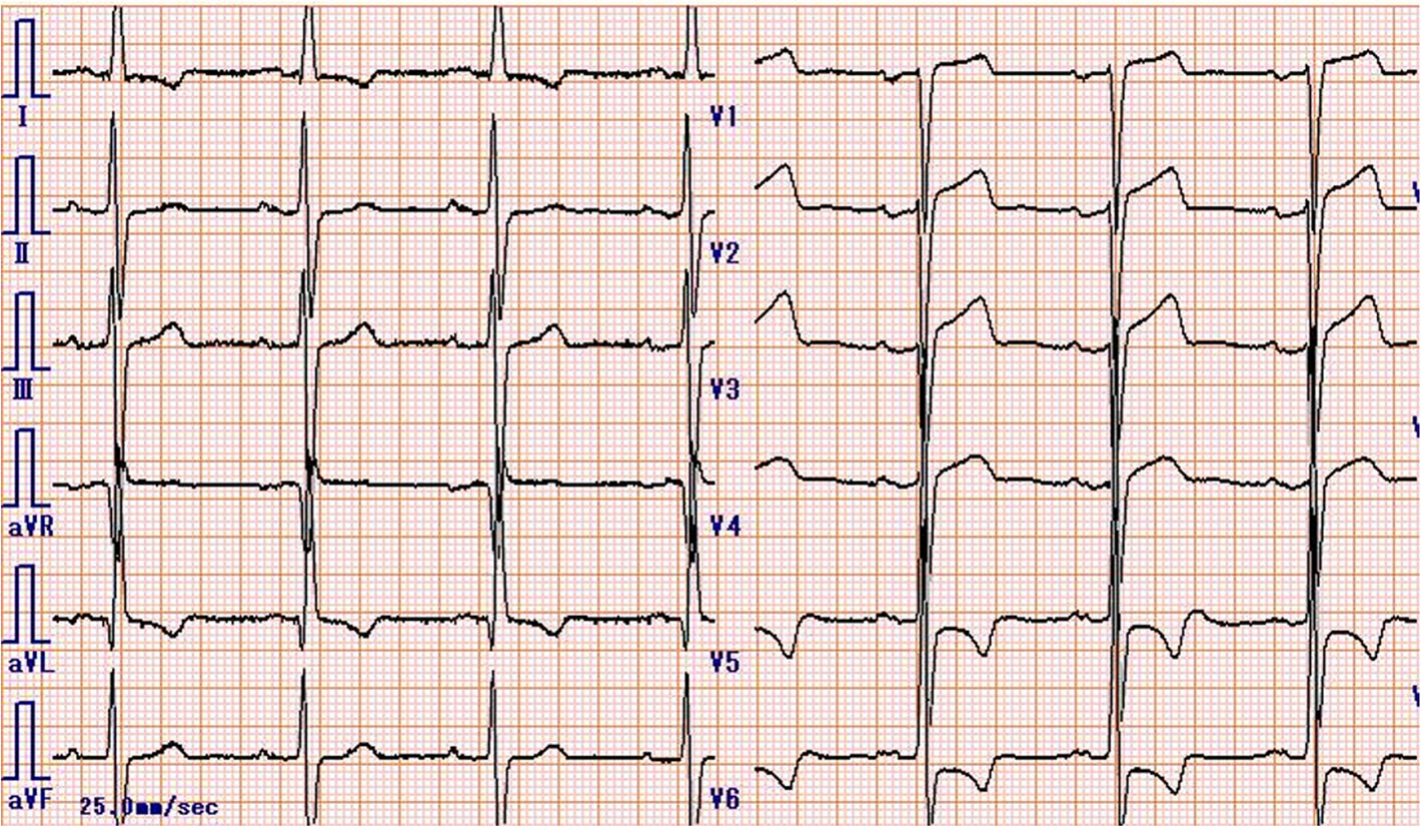
Twelve-lead electrocardiogram showing left ventricular hypertrophy with nonspecific ST-T wave changes

**Fig 3:**
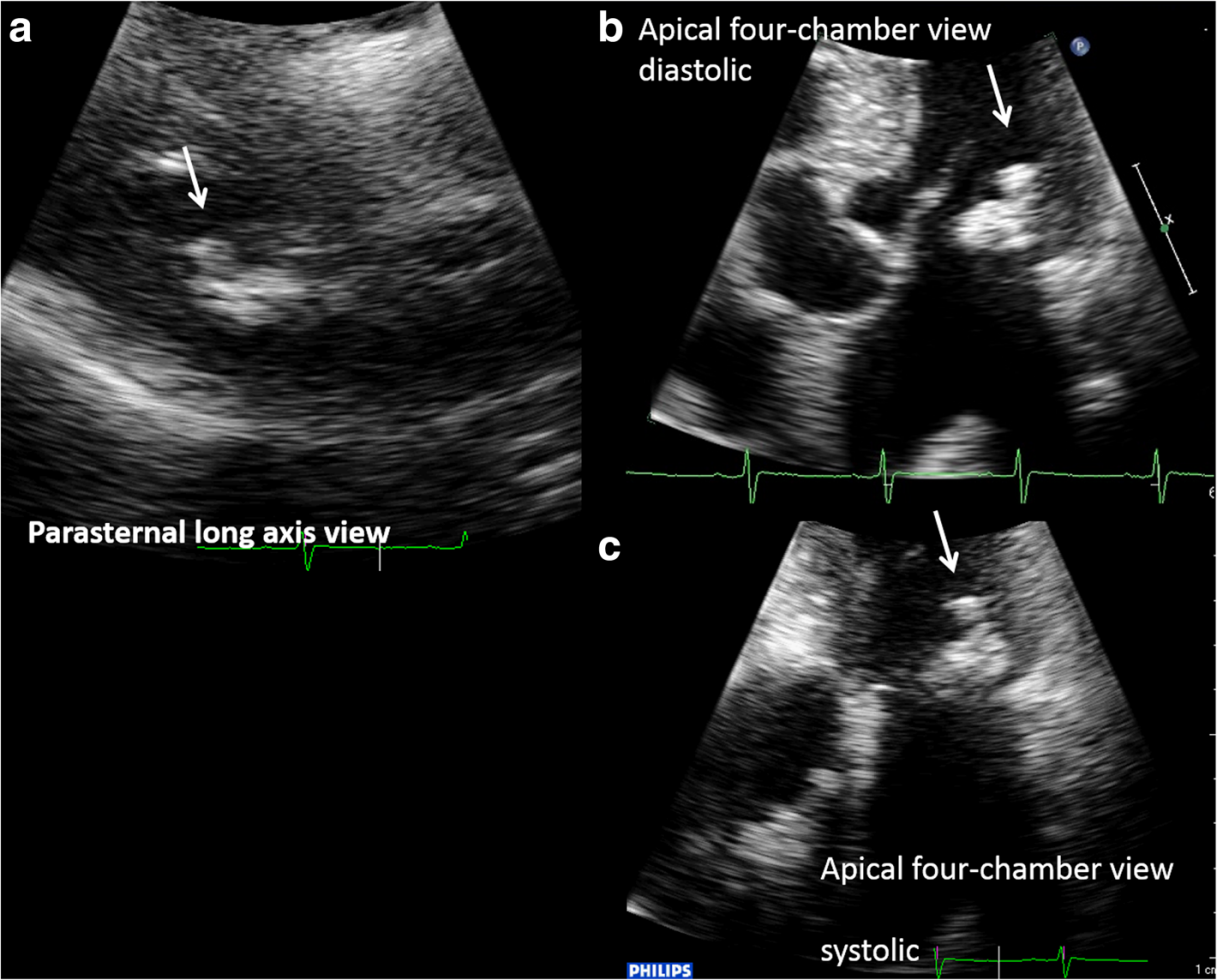
Transthoracic echocardiography. **a** Parasternal long axis view. **b** Apical four-chamber view (diastolic). **c** Apical four-chamber view (systolic). Transthoracic echocardiography revealed a highly mobile echogenic mass (*white arrow*), 8 × 6 mm, attached on the ventricular side of mitral annulus, with severe calcification

**Fig. 4:**
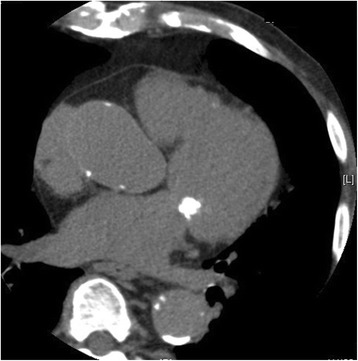
Cardiac computed tomography demonstrated a calcified cardiac mass in the mitral annulus with heavy mitral annular calcification

A chest X-ray showed mild cardiomegaly without pulmonary congestion (Fig. 1). An electrocardiogram suggested left ventricular hypertrophy with nonspecific ST-T wave changes (Fig. 2). A transthoracic echocardiogram revealed MAC and a highly mobile mass (8 × 6 mm) attached to the ventricular side of the posterior mitral valve leaflet (Fig. 3). The echogenicity of the mass was similar to MAC. The mitral valve was mildly thickened with mild mitral stenosis. Left ventricular systolic function was normal. Cardiac computed tomography demonstrated a calcified cardiac mass in the mitral annulus with heavy MAC (Fig. 4). As the mass was highly mobile, suggesting a high risk of embolization, he underwent surgical resection of the mass. Intraoperative findings demonstrated a severely calcified mitral annulus and attachment of the calcific mass to the P2 segment of the mitral valve. Surgical resection was performed successfully.

**Fig. 5:**
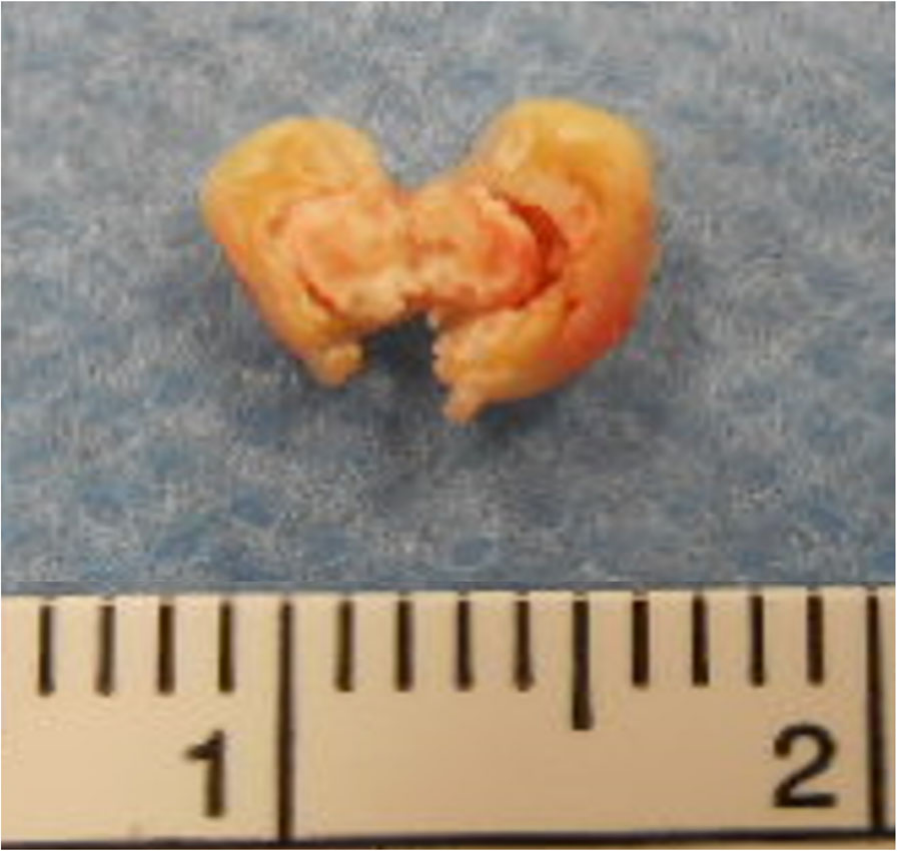
Macroscopic findings of the resected mass showed a *yellowish* color and a partially calcified mass (7 mm diameter)

**Fig. 6:**
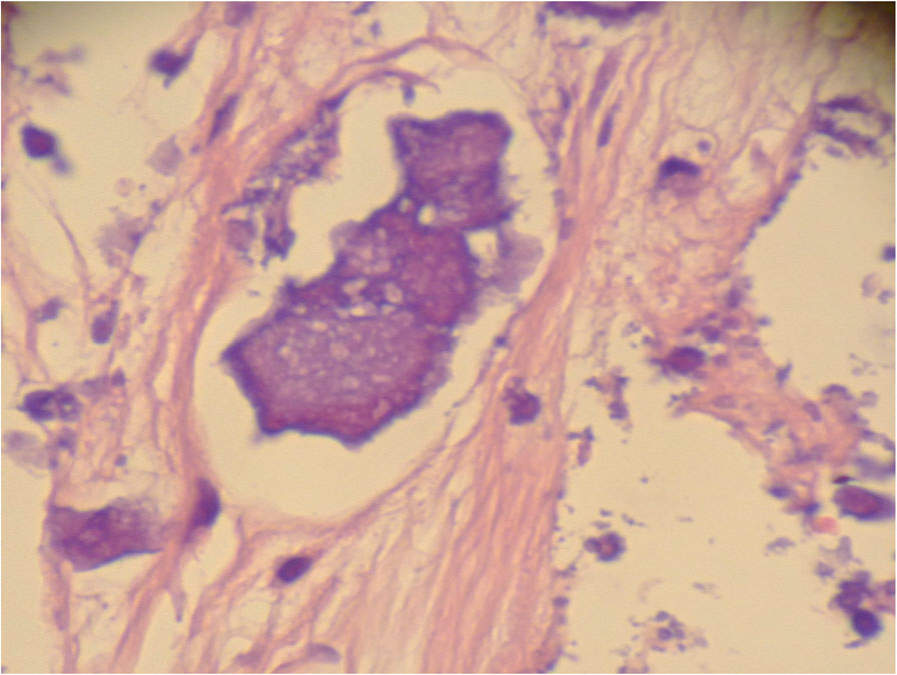
Histopathological examination revealed multiple nodular amorphous calcifications and fibrous connective tissue. There were no identifiable myxoma or malignancy cells. Consequently, the diagnosis of calcified amorphous tumor was confirmed

Macroscopic findings of the resected mass showed a yellowish colored and partially calcified mass (7 mm in diameter; Fig. 5). A histopathological examination revealed multiple nodular amorphous calcifications and fibrous connective tissue. There were no identifiable myxoma or malignancy cells (Fig. 6). Consequently, the diagnosis of CAT was confirmed. He recovered uneventfully and was discharged without any complications.

No evidence of tumor recurrence was detected after the surgery, and he is doing well after 6 months of follow-up.

## Discussion

CAT is a rare non-neoplastic cardiac mass. Although the origin of CAT is unclear, approximately 20% of patients with CAT have ESRD [3], suggesting a potential role of phosphocalcic metabolism abnormalities [4]. In addition, hypercoagulable state was reported to be a risk factor of CAT without ESRD [5]. CAT is defined by pathological findings of calcified nodules in an amorphous background of fibrin, with degeneration and focal inflammation [1]. It is often difficult to distinguish CAT from other cardiac tumors, vegetation, and thrombus when using single imaging modalities. Most patients with CAT undergo surgical resection due to the high risk of embolization, which remains the diagnostic and therapeutic standard [6]. The sizes of CATs were recently reported to range from several millimeters to 90 mm [3]. Patients with CAT are mostly asymptomatic at presentation, although the masses can cause symptoms related to obstruction or embolization, such as dyspnea and syncope [3].

In the present case, the CAT arose from MAC. Previous cases of MAC-related CAT have been reported (Table 1), and this entity should be considered in a differential diagnosis. Of importance, all of those cases apart from one had ESRD [7].

**Table 1:** Review of mitral annular calcification-related calcified amorphous tumors

Case	Authors	Age/Sex	Site	Symptoms	Size (mm)	ESRD	Treatment
1	Kawata *et al*. [4]	59/M	MA	None	6 × 28	+	Resection
2	Fujiwara *et al*. [9]	58/M	MA	None	N.A.	+	Resection/MVP
3	Fujiwara *et al*. [9]	65/M	MA	None	N.A.	+	Resection
4	Kubota *et al*. [6]	64/F	MA	None	3 × 27	+	Resection/MVR
5	Kubota *et al*. [6]	44/M	MA, LV, LA, PM	None	5 × 28	+	Resection
6	Nishigawa *et al*. [7]	78/F	MA	None	N.A.	-	Resection
7	Our case	70/M	PM	Chest discomfort	6 × 7	+	Resection

*ESRD* end-stage renal disease, *F* female, *LA* left atrium, *LV* left ventricle, *M* male, *MA* mitral annulus, *MVP* mitral valve plasty, *MVR* mitral valve replacement, *N.A.* not available, *PM* posterior mitral valve leaflet

+ means presence, - means absence

Further, MAC-related CAT had a highly mobile characteristic, with a high risk of stroke or other systemic embolism. Finally, all of those cases had received surgical resection. Thus, because of the high risk of cardiovascular events in patients with MAC alone [8], MAC-related CAT should be regarded as distinct from other immobile CATs. Our case was considered to have a high risk of embolization. Of note, CAT is a non-neoplastic cardiac mass, and there are no reports of metastasis and death related to CAT. However, postoperative recurrence of CAT due to incomplete resection has been reported [5]. Thus, careful clinical and echocardiographic follow-up is required after surgery.

## Conclusions

We reported a case of MAC-related CAT. In general, MAC-related CAT is considered a high risk for stroke or other systemic embolism. However, it is difficult to diagnose CATs without pathological findings. Therefore, surgical therapy remains the optimal approach for treatment of CATs.

## Abbreviations

CAT: Calcified amorphous tumor
ESRD: End-stage renal disease
MAC: Mitral annular calcification
